# SynFind: Compiling Syntenic Regions across Any Set of Genomes on Demand

**DOI:** 10.1093/gbe/evv219

**Published:** 2015-11-11

**Authors:** Haibao Tang, Matthew D. Bomhoff, Evan Briones, Liangsheng Zhang, James C. Schnable, Eric Lyons

**Affiliations:** ^1^Center for Genomics and Biotechnology, Fujian Agriculture and Forestry University, Fuzhou, Fujian Province, China; ^2^School of Plant Sciences, iPlant Collaborative, University of Arizona; ^3^Department of Agronomy and Horticulture, University of Nebraska, Lincoln

**Keywords:** synteny, homology, genome evolution, cyberinfrastructure

## Abstract

The identification of conserved syntenic regions enables discovery of predicted locations for orthologous and homeologous genes, even when no such gene is present. This capability means that synteny-based methods are far more effective than sequence similarity-based methods in identifying true-negatives, a necessity for studying gene loss and gene transposition. However, the identification of syntenic regions requires complex analyses which must be repeated for pairwise comparisons between any two species. Therefore, as the number of published genomes increases, there is a growing demand for scalable, simple-to-use applications to perform comparative genomic analyses that cater to both gene family studies and genome-scale studies. We implemented SynFind, a web-based tool that addresses this need. Given one query genome, SynFind is capable of identifying conserved syntenic regions in any set of target genomes. SynFind is capable of reporting per-gene information, useful for researchers studying specific gene families, as well as genome-wide data sets of syntenic gene and predicted gene locations, critical for researchers focused on large-scale genomic analyses. Inference of syntenic homologs provides the basis for correlation of functional changes around genes of interests between related organisms. Deployed on the CoGe online platform, SynFind is connected to the genomic data from over 15,000 organisms from all domains of life as well as supporting multiple releases of the same organism. SynFind makes use of a powerful job execution framework that promises scalability and reproducibility. SynFind can be accessed at http://genomevolution.org/CoGe/SynFind.pl. A video tutorial of SynFind using *Phytophthrora* as an example is available at http://www.youtube.com/watch?v=2Agczny9Nyc.

## Introduction

Conserved synteny refers to an inferred homology relationship between genes which are supported by sharing a common genomic neighborhood, and is a widely used measurement of evolutionary divergence across all domains of life (Moreno-Hagelsieb et al. 2001; Engstrom et al. 2007; Heger and Ponting 2007; Poyatos and Hurst 2007; Tang, Bowers, et al. 2008). Conserved synteny is evident when large sets of genes or genomic features are preserved in close proximity (synteny), and often in the same order and orientations (colinearity) (Tang, Bowers, et al. 2008). Conserved synteny across species lays an essential foundation for genomic research, including map-based cloning, validating predicted gene models (Law et al. 2015), and identifying conserved noncoding sequences (Haudry et al. 2013). Conserved synteny within species identifies ancient polyploidy events or other types of large-scale genomic duplications (Wolfe 2001).

Synteny provides an extra layer of information to confirm gene homology, and is much more reliable than inference based on sequence similarities alone. Results from a typical Basic Local Alignment Search Tool (BLAST) analyses do not easily indicate whether there is a gene loss or transposition. Popular approaches based on the reciprocal best hit do not take into account the ancestral state of a genome nor provide much insight into the evolutionary history of a gene or gene family. More generally, protein clustering algorithms such as OrthoMCL (Li et al. 2003) and INPARANOID (Ostlund et al. 2010) may be successful for single copy gene families when evolutionary rates are constant, but can be confounded by accelerated rates of evolution in certain gene copies, and will sometimes produce false-positive assignments of orthology, particularly in cases of reciprocal loss of paralogous genes between species. Positional studies that track gene movements over evolutionary time require more gene-centric synteny tools (Woodhouse et al. 2011).

Curated syntenic gene sets are critical tools for deriving genome-scale patterns and evolutionary trends, and are widely popular (Woodhouse et al. 2011; Baxter et al. 2012; Schnable et al. 2012). Unfortunately, construction of robust and accurate syntenic data sets requires a set of specialized comparative genomic skills currently limited to a small number of research groups. Until now, the primary method by which the broader research community employed syntenic information in their research is through manually curated syntenic gene sets published by these groups. Manually curated gene sets are inherently limiting because, as a result of the lag introduced by the publication cycle, by the time a given syntenic gene set is published, genome assemblies for new species will often have become available, and genome assemblies, annotations, and gene identifiers will often have been updated for existing published genomes. Genome sequence assemblies being released at an ever increasing pace, there is a need for tools that enable individual researchers to rapidly identify syntenic regions between species.

The majority of community use of synteny data generally falls into one of several use cases: 1) Researchers interested in a specific gene from a specific species who want to rapidly find the syntenic ortholog(s) of their target gene in one or more additional species and 2) researchers who want to trace changes in the positional history of a single gene or gene family across a population of related species. In addition to the lag time introduced in publishing syntenic gene lists, most published lists only provide information on conserved syntenic orthologs, but do not provide information on predicted syntenic locations for genes where no syntenic orthologs are found. This severely limits their utility for use case #2 above, as it strips out one of the key advantages of syntenic analysis, the ability to identify confident sets of “true negatives.” True negatives include both lineage specific, recently inserted genes (also known as the “gray genome”) (Freeling et al. 2008), and genes conserved at syntenic locations across multiple species in a clade but deleted from the genomes of one or more specific species. Many evolutionary studies require the knowledge of whether a certain gene is indeed missing or relocated from a genomic region (transposition). Distinguishing transposition from gene removal is critical because potential changes in gene expression patterns are different under these two scenarios.

Identification of syntenic genes has additional advantages for functional research studies, as syntenic homologs are more likely to retain the same expression pattern than nonsyntenic homologs (Dewey 2011; Schnable 2015). Orthologous genes (as identified by OrthoMCL) at nonsyntenic locations show reduced correlation in expression pattern between different grass species (Davidson et al. 2012). Genes captured by helitrons and relocated to a new genomic neighborhood in maize show novel patterns of expression (Barbaglia et al. 2012). Common methods of gene transposition—transposon capture (Lai et al. 2005) and intrachromosomal recombination (Woodhouse et al. 2010)—can often carry protein-coding sequence of a gene without the associated regulatory sequences. A study in maize also found that genes that retain in syntenic positions across multiple grass species were significantly more likely than nonsyntenic genes to produce visible mutant phenotypes when knocked out (Schnable and Freeling 2011), further highlighting the functional relevance of synteny information in the validation of direct functional homologs.

As we provide a novel implementation of yet another synteny-finding tool, we offer an overview of popular synteny-finding algorithms, including several tools that were designed and implemented by several of the authors in the past. In general, the synteny-finding algorithms can be grouped based on whether they are based on positional colinearity or positional density, for what type of statistical features they are searching (Ghiurcuta and Moret 2014), and their definition of “syntenic block.” A list of recent synteny search software includes iAdHore (Proost et al. 2012), mGSV (Revanna et al. 2012), SyMap (Soderlund et al. 2011), SynMap (Lyons et al. 2008), Orthocluster (Vergara and Chen 2010), Synorth (Dong et al. 2009), MCScan (Tang, Wang, et al. 2008), and MCScanX (Wang et al. 2012) among many others. These synteny search software vary greatly in the trade-offs accepted by the authors in terms of run time, computational resource requirements, and goal of minimizing either type I (false positive) or type II (false negative) errors. In addition, from a pragmatic standpoint, the tools are also distinguished by interface type (i.e., command line, web based) and whether a given tool offers the built-in functionality to provide graphical outputs, enabling visual proofing of results. Herein, we provide a review of major features of recent synteny-finding software in table 1.

**Table 1:** Comparison of Major Features of Synteny-Based Homology Detection Software

Tool	References	Interface	Multiple Genomes	Syntenic Families	Infer Gene Loss	Scoring Mode	Parallel Computing	Integration with Data
ColinearScan	Wang et al. (2006)	Command	−	−	−	Colinear	−	−
Cinteny	Sinha and Meller (2007)	Web	+	−	−	Colinear	−	Limited (∼20)
MCScan	Tang, Bowers, et al. (2008)	Command	+	+	−	Colinear	−	−
SynMap	Lyons et al. (2008)	Web	−	−	−	Hybrid	−	CoGe (∼25K)
MCMuSeC	Ling et al. (2009)	Command	+	+	+	Synteny	−	−
OrthoClusterDB	Ng et al. (2009)	Web	Limited	−	−	Colinear	−	Limited (∼50)
Cyntenator	Rodelsperger and Dieterich (2010)	Command	+	−	−	Colinear	−	−
MicroSyn	Cai et al. (2011)	GUI	+	+	−	Synteny	−	−
SyMAP	Soderlund et al. (2011)	GUI/Web	+	−	−	Hybrid	−	Limited (∼10)
MCScanX	Wang et al. (2012)	Command	+	+	−	Colinear	−	−
i-ADHoRe	Proost et al. (2012)	Command	+	+	−	Both/Hybrid	+	−
SynFind		Command/Web	+	+	+	Both	+	CoGe (∼25K)

Note.—The tools published in the last 10 years are given in the table. Symbols + and − represent yes and no, respectively. “Scoring mode” is the optimization goal used in identifying syntenic regions. “Colinear” requires the gene order to be preserved; “Synteny” does not enforce conserved gene order; “Hybrid” uses “Colinear” initially and recruits imperfect synteny; “Both” supports both modes as program options. “Integration with data” is a count of available genomes for immediate use with a given tool.

A careful evaluation of these algorithms suggested fundamental challenges that are still not met for more general uses. First and foremost, data curation is often a significant challenge (Lohr 2014), requiring users to convert genomic annotation files into a range of idiosyncratic file formats required by different algorithms. Many tools are run from the command line, and often obtaining the most accurate results from a given tool will require experimentation with a range of settings, presenting an additional challenge to users who must develop methods of evaluating and ranking multiple output data sets. As the number of organisms a user is interested in comparing grows, computational time requirements will often scale quadratically, presenting challenges for these primarily offline algorithms.

After closely working with researchers in the community in the past few years, it was clear that the life cycle of gene synteny analysis requires running multiple algorithms to create input homology data (different BLAST-like algorithms), adjusting parameters on-the-fly (configurable thresholds), as well as allowing different synteny-finding/scoring schemes (colinear vs. density) (table 1). Following the same design principle as other CoGe tools, we continue to adopt a cloud-based implementation that offers a one-stop solution that combines user-configurable input data (genomes and structural annotations), algorithms, scalable computing resources (parallelization, memory, and storage), integrated visualization, links to additional tools for further data analysis, readily exportable results, and reproducibility through permanent URLs.

Our new online method, SynFind, has a number of features not typically found in other systems (table 1) that reflect recent innovations in comparative genomic analysis adopted in a few newly sequenced genomes (Amborella Genome Project 2013; Ibarra-Laclette et al. 2013; Chalhoub et al. 2014; Green et al. 2014). SynFind identifies multiple syntenic regions between a gene in a reference genome and a target genome, entirely independently of whether syntenic ortholog or paralog is present at the predicted location or not. SynFind provides the option for both density and colinear scoring of syntenic regions to address the different structural genomic changes in taxa with different evolutionary distances and different genome assembly qualities. SynFind generates syntenic depth tables as well as gene presence–absence table to reveal ancient polyploidy events and genes unique to one genome against others. Most critically, the integration with CoGe provides instant access to thousands of genomes across all domains of life along with CoGe’s tools to let users add new genomes, keep them private, and compare them using SynFind as rapidly as they are released. Tight integration with up-to-date genomic data facilitates access to computing resources, downstream visualization and analysis tools, thereby creating an open-ended pipeline of research that facilitates exploration of multidimensional genomic data sets that bridge evolutionary genomics and functional genomics.

## Materials and Methods

### Synteny Score

SynFind processes putatively homologous gene pairs in order to extract the syntenic blocks, using each gene as query. Gene pairs are computed from sequence similarity search programs, such as BLAST, LASTZ, or LAST (Kielbasa et al. 2011). The modular architecture of SynFind allows the straightforward incorporation of new sequence similarity search algorithms in the future. Although SynFind can output information for a single gene, in each run, syntenic regions in the target genome(s) are identified for every annotated gene in the query genome. Extra caution is taken with genes which are members of tandem arrays (groups of homologous genes clustered together in the genome) as matches among such genes are likely overcounted and show up as false-positive synteny blocks. Consequently, tandem matches are reduced to a single copy in this step to avoid seeding a synteny block inside a tandem array. The treatment of tandem arrays is similar to the strategy used in MCScanX and iADHoRe (Proost et al. 2012; Wang et al. 2012).

**Fig. 1.—:**
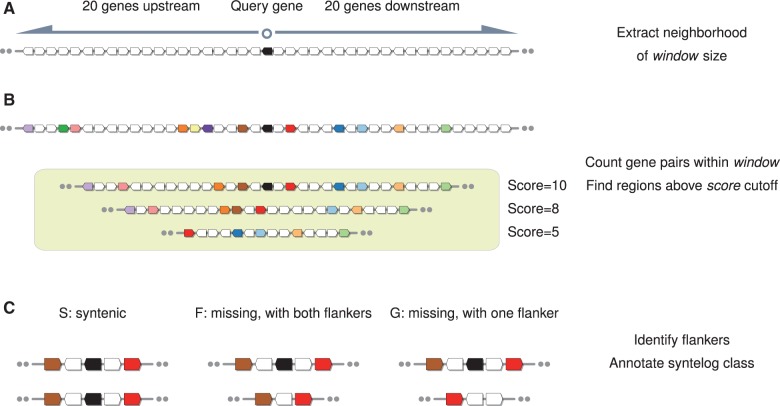
Illustration of three key steps in SynFind. The three key steps include (*A*) extraction of genomic neighborhood, (*B*) gene pair generation and scoring of each matching region, and (*C*) identification of flankers (neighboring gene pairs) and annotation of syntelog class.

To seed synteny blocks, our algorithm works by selecting a fixed number of genes up and downstream from the query gene (fig. 1*A*). This method is robust with respect to variation in gene density and intergenic spacing observed across different species. All gene pairs to a target genome between the region surrounding the gene of interest and candidate syntenic locations in the target genome are then identified and the number of matching gene pairs is counted as the “synteny score” (fig. 1*B*). SynFind provides positioning cues for visualization through genome browsers. Comparisons across sets of homologous regions are facilitated through automated centering and truncation of colinear panels. The middle gene of the current window or the “query” is used to as the center of the syntenic panels. The extent of syntenic gene pairs in the current window can be used to truncate the matching panels to focus on a particular region of interest. Finally, SynFind automatically flips sequences so syntenic regions are visualized on the same strand for clarity. These data are useful in automatically creating local syntenic views in CoGe for subsequent manual validation.

The output of the seeding step consists of syntenic gene pairs and a score to indicate the level of conserved synteny between their respective genomic locations. For each target region found, the synteny score reflects the number of gene pairs that are syntenic or colinear within the window, depending on the scoring function. When a matching region is found, the flanking genes for the query gene are identified and the status of the syntelog is tracked in a single letter notation—S/F/G, following the nomenclature in Woodhouse et al. (2011). S is “syntelog,” which means that it has a match to the region. In this case, the match itself is used to represent the region. In contrast, F class and G class refer to the cases that the syntelog is missing (fractionated or moved) from syntenic region identified in the target genome. F has both flankers present, whereas G has only one flanker (fig. 1*C*). G class syntenic regions are largely the result of adjacent genomic rearrangements (inversions and translocations) in either the target or query genome, but can also occur at the end of pseudomolecules, scaffolds, or contigs. In the case of F or G, a flanker gene is used to represent the region as a “proxy” to identify the approximate location of where a syntelog is expected to reside in the target genome.

As a final validation, we recover tandem matches by checking against the original BLAST output as the tandem matches were reduced to single copy prior to the “seeding” step. This validation step increases the sensitivity of SynFind for genes inside tandem arrays. A single best match among the tandem array is selected to be the representative syntelog for a query gene, for the sake of clarity. The source code of SynFind can be found at https://github.com/tanghaibao/quota-alignment/blob/master/scripts/synteny_score.py (last accessed November 30, 2015).

### Choice of Parameters: Beauty in Simplicity

There are a few intuitive, user-configurable parameters that adjust sensitivity or specificity of SynFind.

#### Window Size: Window Size in Number of Neighboring Genes (Default: 40)

Given an anchor gene, SynFind searches upstream and downstream half a window size from the query. For example, a window size of 40 means that a total of 41 genes are checked: The query gene, plus 20 upstream genes and 20 downstream genes (fig. 1*A*).

*Minimum synteny score*: The minimum number of anchoring genes to call a region “syntenic.”

The combination of “window size” and “minimum number of genes” together controls the sensitivity and specificity of the algorithm (fig. 1*B*). The default number 4 means that a region is considered syntenic if 4 of 41 genes are syntenic. This threshold is capable of finding weakly homologous regions, such as regions undergoing high degree of fractionation following polyploidy. In our test, moving the threshold below 10% would often run into the risk of false positives due to repeats and gene transpositions.

#### Scoring Function

Scoring can be based on colinearity or density. For colinearity, a colinear arrangement of syntenic genes is enforced, based on the “longest increasing subsequence” method (Woodhouse et al. 2011). For density, we use single-linkage clustering to group gene pairs within the window in comparison, and any arrangement of gene-pairs is tolerated. Although colinearity is frequently used in plant genome comparisons, synteny without requiring shared order is often the only criteria in the comparison of insect and vertebrate genomes, due to different rates and scales of inversions and translocations between plant and animal genomes (Tang, Bowers, et al. 2008). The two different scoring functions allow flexibility in accommodating taxa with different modes of karyotypic evolutions.

#### Maximum Syntenic Depth: Limit the Number of Syntenic Regions Up To the Specified Depth

This parameter is useful in lineages with shared duplication events. Enforcing the syntenic depth allows screening of regions derived from specific evolutionary events (Tang et al. 2011). In particular, enforcing a maximum syntenic depth of 1 between species which are diploid relative to each other, but share one or more ancient whole-genome duplications (WGDs) would limit the search to only orthologous regions. The default is to output all syntenic regions found.

### CoGe Implementation

**Fig. 2.—:**
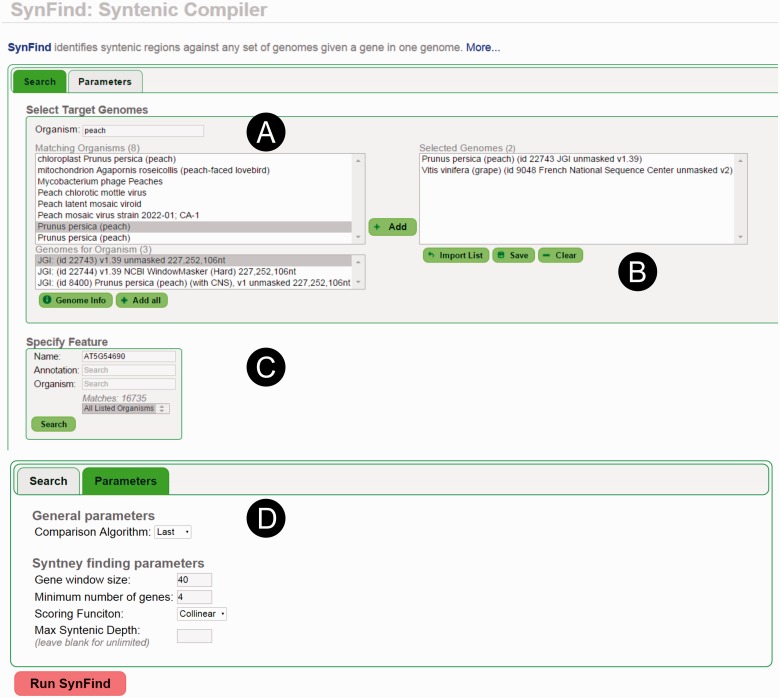
SynFind web UI. The web UI includes several components that users can interact with (*A*) find target genome and select target genome version, (*B*) build list of multiple target genomes, (*C*) input query gene, (*D*) set SynFind parameters.

SynFind is implemented as one of the main entry points and analytical tools of CoGe. The user-interface (UI) contains two sections: One which is used to select a gene of interest and target genomes to search for syntenic homologs, the other to specify SynFind’s algorithms and parameters (fig. 2). This UI is consistent with the general look-and-feel for other CoGe tools. CoGe’s implementation of SynFind allows users to search an arbitrary number of genomes for syntelogs of any gene located in a genome to which the user has access. Specifically, the genomes need to be any public data sets or private data sets that are owned by or shared with the user. Target genomes to be analyzed by SynFind are similarly specified by searching for organisms by name or taxonomic description, and then selecting the appropriate genome (fig. 2*A*). By repeating the name searches, several genomes may be added to the genome list (fig. 2*B*). Researchers may also select a previously saved genome list (e.g., a list of “ten grass genomes that have been sequenced thus far”) as a shortcut for researchers interested in a frequently accessed set of species. SynFind depends on the existence of structurally annotated protein coding gene models as a starting point for any query (fig. 2*C*). Some “draft” genome assemblies are released and loaded into CoGe with no available gene annotations. These genomes are automatically detected and excluded from the genome list (with information presented to the user as to why the genome is blocked from analysis by SynFind). In the configuration tab, users can select which algorithm to use for generating the homology pairs file as well as SynFind parameters: Window size, minimum number of genes to call a region syntenic, and the scoring scheme (colinear or density) (fig. 2*D*).

**Fig. 3.—:**
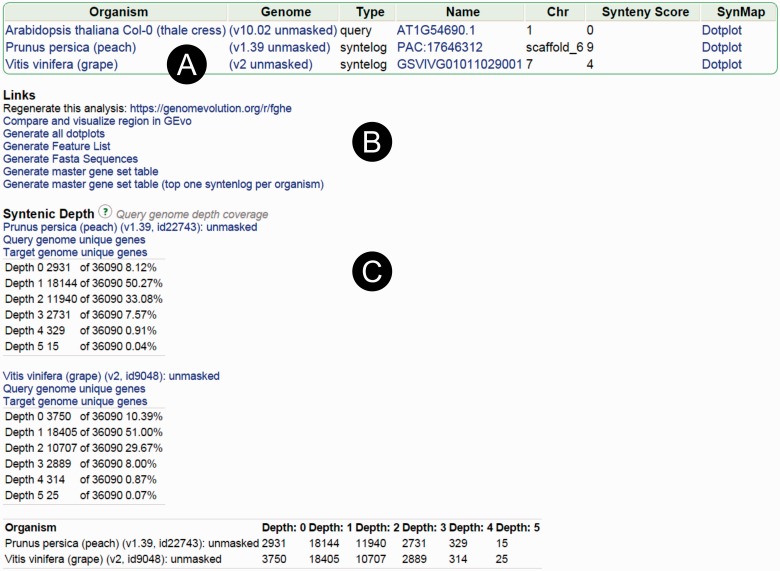
SynFind example output. The output of a typical SynFind search: (*A*) List of all syntenic regions found and presence of syntelog, (*B*) links for micro-synteny viewer (GEvo) and master tables for downstream analyses, (*C*) syntenic depth table useful for evaluating syntenic coverage and WGD events.

When SynFind completes its analysis, the results show a table of matching regions along with their synteny scores and whether or not a syntenic gene was identified (fig. 3*A*). Additional links are available under the table, including microsynteny analysis of the identified regions in GEvo for validation, pairwise syntenic dotplots in SynMap, links to raw data and intermediate data files, and a link to revisit and regenerate the same SynFind analysis (fig. 3*B*).

### Master Syntenic Pairs Table

SynFind identifies syntenic regions against any set of genomes given a gene in one genome, and curates the results in a master gene list. The pan-genome master list is important as this file contains all the syntenic regions identified in the target genomes for all of the genes in the query genome. The master list is a tab-delimited table, containing all syntenic gene sets between the query and target genomes, along with links to visualize microsynteny for each local set of region. As a filtering option, SynFind can also report top *N* best matches in query genome(s), which is useful to extract only orthologous regions that are often the best syntenic match when *N* is set to 1. As a byproduct of this master gene pairs table, SynFind reports a list of genes that are unique to some genomes. For example, in the case of comparing a set of bacterial strains, this feature can be used to find pathogenicity genes and phage insertions specific to one strain against others (Tettelin et al. 2005).

### Syntenic Depth

Syntenic depth refers to the number of syntenic regions identified in a target genome for a given query position. SynFind calculates syntenic depth on a per gene basis and reports these data as a histogram, showing a breakdown of how many genes are covered in 1-, 2-, to *x*-fold regions (fig. 3*C*). Genes with a syntenic depth of zero are the genes that lack any matching region in the target genome. A syntenic depth of one most often reflects identification of an orthologous genomic region between two species, whereas a syntenic depth greater than 1 most often is the result of either paralogous or co-orthologous regions derived from whole-genome (or other large scale) duplications. Syntenic depth provides a more consistent marker for large scale genomic events than changes in the copy number of individual genes which are influenced by a greater number of small scale processes (expansion and contraction of tandem arrays, transposon capture and duplication, etc.). The proportion of genes with a syntenic depth of at least 1 is a useful metric for evaluating the relative completeness of genome assemblies, whereas modal and maximum syntenic depths are good indicators for the number of paleopolyploidies in a given lineage.

Plant genomes have rich history of genome-wide duplication events that give rise to very high level of syntenic depth (Tang, Bowers, et al. 2008). For example, in comparison to *Arabidopsis* genome, both peach and grapevine genomes show significant genome coverage of depth up to 3 (fig. 3*C*), corresponding to the pan-rosid genome triplication event (Lyons et al. 2008; Tang, Bowers, et al. 2008). The syntenic depth evaluation of SynFind was employed to identify multiple degenerate polyploidy events in the highly compact plant genome, Utricularia (Ibarra-Laclette et al. 2013). Examples of various syntenic depth tables and their interpretation in the context of paleopolyploidy can be found on CoGePedia (http://genomevolution.org/r/4suf, last accessed November 30, 2015).

## Results and Discussion

### Focused Analyses for Functionally Important Genes

We show that SynFind is powerful for gene-centric analyses through selected examples based on past studies, but the usage is generally applicable to almost any gene family members in any set of organisms available in the CoGe database. In the past, such comparative analyses would usually take much dedicated time and work—from downloading and reformatting data sets, performing sequence alignment, reformatting data again for use in synteny detection tools, identifying syntenic genes, selecting informative visualization software for manual validation, and performing multiple analyses to identify an optimal configuration of parameters and software tools—all of which can now be performed within the SynFind tool in a few clicks.

One natural application of SynFind is to deduce gene presence and absence across a set of related organisms. In the context of bacterial genomics, we can infer possible pathogenic sequences through syntenic comparisons (Jin et al. 2002; Tettelin et al. 2005). We used SynFind to compare three-way *Shigella flexneri* 2a strain 301, *Escherichia coli* K12 substrain 1655 and *Escherichia coli* O157:H7 strain EDL933, in an analysis similar to the study in Jin et al. (2002). When using *S. flexneri* genome as the query, we looked for the cases where SynFind reported either proxy in the two *E. coli* genomes, that is, the genes that were missing in their expected locations or for which expected regions could not be identified. This has allowed us to identify *Shigella-*specific “islands.” In particular, one 27 gene island (from *SF0294* to *SF0320*) found only in the *Shigella* genome, previously termed SfII, was shown to be a lysogenic phage insertion, by which *Shigella* might have acquired virulence (Jin et al. 2002). Other interesting genes on these *Shigella*-specific islands include *ipaH* genes (e.g., *SF0722*, *SF1383*, *SF1880*, and *SF2610*) that shared homology with different phages (Jin et al. 2002). The SynFind link to this analysis is available: https://genomevolution.org/r/fggo (last accessed November 30, 2015).

**Fig. 4.—:**
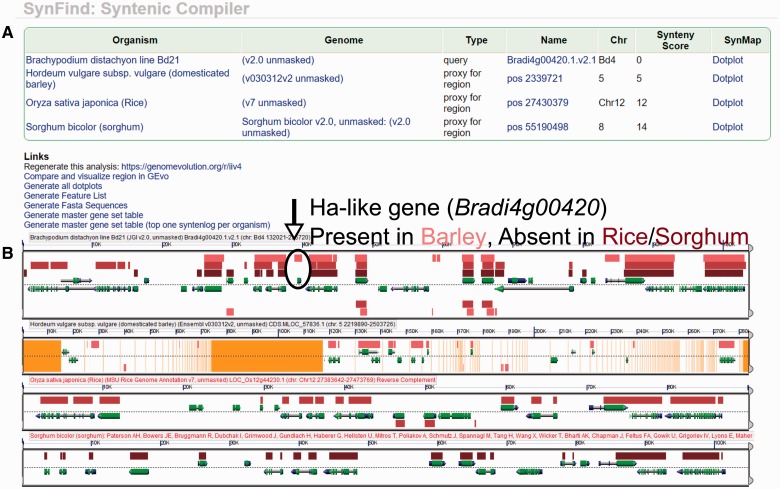
SynFind analysis of *Ha*-like gene across Brachypodium, barley, rice, sorghum. (*A*) SynFind table output illustrating four matching regions in the selected grasses. Result can be regenerated: https://genomevolution.org/r/iiv4 (last accessed November 30, 2015). (*B*) GEvo visualization of the compiled syntenic regions, showing the presence of a syntenic sequence in barley, and lack of syntenic ortholog in *Ha*-like gene in rice and sorghum. Each panel represents a syntenic region in Brachypodium, barley, rice, and sorghum, from top to bottom. Arrows in each panel represent gene models, and boxes on top of the gene models are sequence matches (HSPs). For the top Brachypodium panel, there are three tracks of HSPs, which are to barley, to rice and to sorghum, respectively. We can conclude that the *Ha*-like gene in Brachypodium has match to barley and no match to rice and sorghum. Result can be regenerated: https://genomevolution.org/r/iivx (last accessed November 30, 2015).

As our second example, we use another previously studied gene involved in the soft grain trait in the grasses. Genes involved in the soft grain trait has been studied extensively in wheat, including the *Hardness* (*Ha*) locus and several *Ha*-like genes (Charles et al. 2009). SynFind analysis (Brachypodium genes as “query,” barley, rice, and sorghum as “target”) showed that *Ha*-like genes were present in Brachypodium representing the lineage of Pooideae, but were missing in rice and sorghum. For barley, rice and sorghum, SynFind output displays “proxy for region” rather than a direct syntelog (fig. 4*A*). With visual proofing using GEvo, we confirmed that there is a syntenic sequence match in barley, whereas there are no matching sequences in rice and sorghum as indicated by SynFind (fig. 4*B*). This suggested that the flanking regions of *Ha*-like gene were relatively intact whereas the gene itself has been lost in rice and sorghum. Alternatively, the gene could be inserted into this region in Brachypodium and barley. Although both scenarios are equally likely, previous study preferred the scenario that the gene was lost in rice and sorghum (Charles et al. 2009). With SynFind tool, we have confirmed that the presence or absence of the *Ha*-like gene in this set of syntenic regions nicely explains the soft wheat and barley grains versus the hard grains like in rice and sorghum.

In addition to the two examples shown above for the purpose of demonstration, SynFind has enabled a number of evolutionary studies of important functional genes in diverse lineages (Woodhouse et al. 2010; Tang and Lyons 2012; Hofberger et al. 2013; Waters et al. 2013). For example, SynFind was used to screen regions in the *Aethionema arabicum* genome displaying synteny to genomic regions in *Arabidopsis thaliana* harboring glucosinolate biosynthesis (GS) loci (Hofberger et al. 2013). SynFind was essential in clarifying the series of tandem duplication and WGD events that drove GS pathway expansion, which were critical to the evolutionary success to the mustard family (Hofberger et al. 2013). Also, SynFind was essential for proving that the genome of *Utricularia gibba*, despite is small size (82 MB), is derived from three sequential WGD events (Ibarra-Laclette et al. 2013).

### Quality of Homology Assignments and Benchmark of SynFind against Competing Tools

Clade-wide syntenic gene sets are useful for detecting genome-wide transposition and deletion events (Woodhouse et al. 2010; Schnable et al. 2012), and automation of this step could be essential in such studies. We have benchmarked SynFind against a number of studies that typically require a substantial amount of human curation to complete. Although the human curated gene sets are still imperfect and subject to errors, they serve as a basis for comparing between different synteny search tools including SynFind. In this study, we evaluate the performance of SynFind and compare that with competing software including MCScanX and iADHoRe, which are the two most popular state-of-the-art tools that perform well in a number of studies (Proost et al. 2012; Wang et al. 2012).

Our first set of test data is a list of WGD duplicates from *A. thaliana* curated by Bowers et al. (2003). This list contains a total of 5,788 gene duplicates collectively derived from the alpha, beta, and gamma WGDs (Bowers et al. 2003). Our second data set is based on comparison of yeast genomes, using data from Yeast Gene Order Browser (YGOB) (Byrne and Wolfe 2005). We were able to find 14 yeast genomes in the CoGe system, whereas a few yeast species in YGOB were not yet released to GenBank with structural gene annotations and therefore not included in this study. YGOB uses “pillars” to store homology assignments (Byrne and Wolfe 2005), which were converted to gene pairs for validation purposes. Finally, as the third test set, we used a pan-grass synteny gene set curated by Schnable et al. (2012). Schnable et al. manually clustered and curated gene members from rice, Brachypodium, sorghum, and maize according to inter- and intragenomic comparisons (Schnable et al. 2012). A typical set of syntenic genes in the Schnable set contain up to 2 rice genes, up to 2 Brachypodium genes, and up to 2 sorghum genes all derived from the shared pan-grass WGD, and up to 4 maize genes because of an additional maize-specific WGD. Similarly, we converted families into a list of gene pairs before validation. The choice of these data sets is based on the availability of curated data sets, and inclusion of gene sets with both paralogous and orthologous relationships.

**Fig. 5.—:**
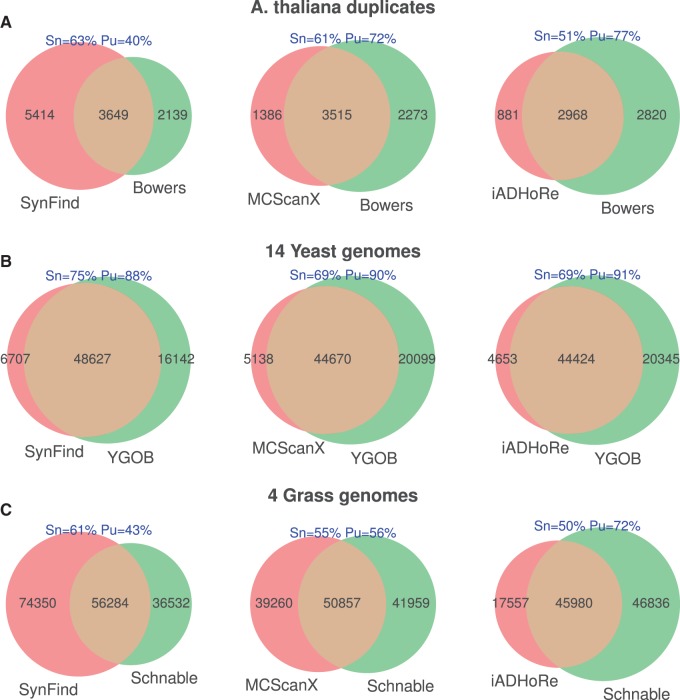
Comparison of SynFind, MCScanX, and iADHoRe on curated data sets. (*A*) *Arabidopsis thaliana* alpha, beta, and gamma duplicates from Bowers et al. (2003). (*B*) Yeast genomes from YGOB (Byrne and Wolfe 2005). (*C*) Grass genomes from Schnable et al. (2012). Sn: sensitivity, defined as common items divided by total items in truth set; Pu: Purity, defined as common items divided by total items in the test set.

For SynFind, MCScanX, and iADHoRe, we computed the syntenic gene list and compared against the curated set, which are considered as “truth” (fig. 5). Two metrics are computed—“Sensitivity” (Sn) is defined as common items divided by total items in truth set; “Purity” (Pu) is defined as common items divided by total items in the test set as can be used to infer false-positive discovery. SynFind consistently ranks the highest in sensitivity, recovering 63%, 75%, and 61% of the items in the truth set (fig. 5). As a tradeoff, the purity of SynFind results compare less favorably than the other tools (fig. 5). As we have designed SynFind as a gene-centric query tool, this benchmark reflects our focus on sensitivity—we would tolerate some false positives but prefer to have low false negatives. Differences in the treatments of tandem gene sets may have contributed to the nonoverlapping members—SynFind, MCScanX, and iADHoRe may have picked a single matching gene within the array which is not necessarily the tandem member in the curated set.

The list of predicted locations for missing genes is often good indication of potential loss-of-function, which could be associated with differences in phenotypic and physiological traits between grasses, as illustrated in our *Ha* example. Missing genes in one grass genome versus others could also suggest possible misassemblies, leading to iterative improvement of genome assemblies and recovery of missing gene fragments in genome annotation efforts (Law et al. 2015).

### Integration with CoGe Comparative Genomics Platform

Integration in CoGe permits SynFind to be tightly connected to thousands of genomes as well as to downstream analysis tools such as GEvo (Lyons and Freeling 2008) and SynMap (Lyons et al. 2008) for micro and whole-genome syntenic analysis, respectively. The method for selecting query and target genomes loads the same module. SynFind automatically generates links to GEvo views for gene-centric analyses as well as SynMap views for chromosome-level analyses. The open-ended analysis workflow provides the users with enough flexibility between tools of different scales. In addition, CoGe’s user-data management systems let researches add private genomes and share them with collaborators, create lists (notebooks) of genomes that can be imported quickly into SynFind, and automatically record links to regenerate any analysis performed.

**Fig. 6.—:**
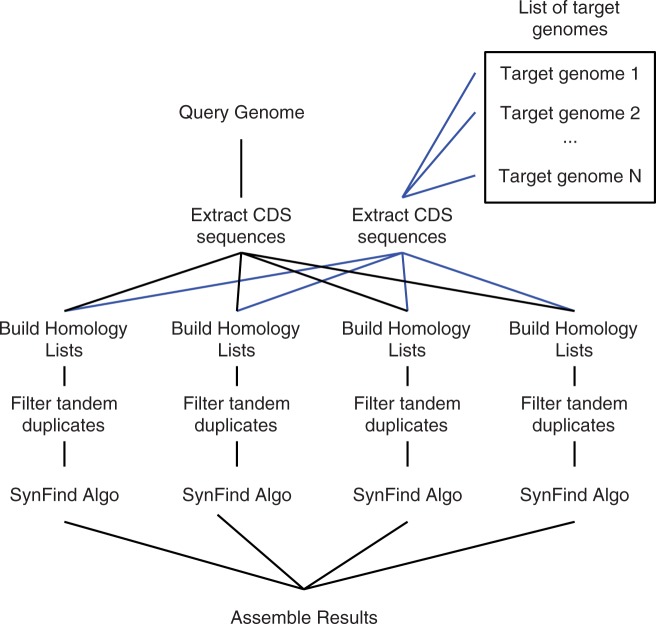
SynFind computational workflow as implemented on CoGe. The query genome and target list of genomes are processed in parallel—extracting coding sequences, building homology lists, filtering tandem repeats, and running SynFind algorithm. The last step assembles the processed data into a master table. This strategy is similar to the “Map-Reduce” paradigm used in parallel computing.

The CoGe job execution (JEX) framework facilitates parallel processing of queries against multiple genomes by using Work Queue (Thrasher et al. 2012) (fig. 6). When a SynFind analysis runs, each pairwise workflow consisting of separate query-target genome pairs is submitted to CoGe’s JEX framework. The JEX framework controls the parallel computing in processing multiple genomes (fig. 6). It first checks to see whether the anticipated results file already exists and retrieves that file if it does, otherwise, it submits the analysis for processing and subsequently caches the results file. This system permits reusing the results of previously run analysis as well as running multiple workflows in parallel. For example, in contrast to other gene clustering approaches, new genomes can be incrementally added to the target list and the CoGe server would only need to compute the missing comparisons. Overall, this greatly improves the performance of the system in terms of the time it takes to complete an analysis. Additionally, if a user decides to modify and rerun an analysis, recomputation starts from the first divergent step of the analysis, while reusing data from earlier, identically configured steps, allowing fast tweaking of parameters.

The scale of analysis in comparative genomics is an important issue. Although SynMap excels in identifying large-scale structural similarities, it lacks the gene-centric searches where researchers just want to study their genes of interest across a set of genomes. This conceptual difference was often referred to as “macrosynteny” versus “microsynteny” analyses in comparative genomics. Microsynteny search tools, such as SynFind, achieve higher sensitivity and more flexibility for gene-centric research. Although SynMap is necessarily constrained to making pairwise comparisons between genomes, SynFind can simultaneously launch comparisons of multiple genomes. Additionally, SynFind identifies syntenic locations even when the gene itself is absent, either as a result of lineage-specific gene deletion or lineage-specific gene insertion. Analyses based on SynMap output required substantial customized offline postprocessing and analysis to generate equivalent predicted locations (Schnable et al. 2012). Importantly, both of these tools permit on-the-fly analyses and allow direct manipulation of parameters (e.g., higher or lower stringency, such as window size and “score cutoff”), and are interconnected in order to characterize and validate patterns of genome structure and dynamics.

A typical exploratory workflow that we recommend would be to 1) use SynMap to characterize genome-wide rearrangements and possibly genome duplications, 2) zoom-in on a pair of contigs or chromosomes with interesting rearrangement or duplication pattern, 3) select a gene to fish out additional syntenic regions using SynFind, and 4) validate putatively syntenic regions using GEvo to ensure that each region covered the entire region of interest. In real-world applications, the combination of SynFind and SynMap can both be applied to offer complementary views. For example, in a study of conservation of imprinting across a set of grass taxa, gene-level comparisons were made between syntenic genes in the genomes of maize, rice, and sorghum using the software SynMap followed by SynFind to offer the most coverage (Waters et al. 2013).

### Scalable and Sustainable Infrastructure for Gene-Centric Evolutionary Study

The SynFind algorithm addresses important limitations and challenges in the postgenomics era. Researchers have access to large and inexpensive sequencing power making it possible to study genetic and genomic evolution across whole clades of species rather than being confined to individual model organisms. However, in order to unlock the potential power of comparative genomic approaches to accelerate studies of the origin, regulation, and function of individual genes it is necessary to enable the broadest possible range of scientists to make direct comparisons across the genomes of large groups of related species. Online computational resources, such as CoGe, create ecosystems of specialized applications that are easily linked to and from one another. Similarly, resources developed by cyberinfrastructure projects such as the iPlant Collaborative (Goff et al. 2011) and XSEDE provide computational platforms that enable scalable access to computing and data storage resources.

The development of computational ecosystems which will be successful in bringing about a democratization of bioinformatics research requires the deployment of modular analysis pipelines that allow each new tool to exploit existing computational resources, architectures, and curated data sets. SynFind joins the increasing list of CoGe-powered and iPlant-enabled applications (Goff et al. 2011), which already include GEvo, SynMap, and many others. The availability of SynFind will begin to merge the two analytical worlds of comparative and functional genomics such that researchers can more easily transfer system-level functional knowledge from data-rich model organisms to the thousands of others organisms being analyzed by only a handful of scientists. Conversely, SynFind enables comparative, in silico studies across a wide range of species to inform the study of specific genes within model organisms, where even today 30–34% of all genes have no annotated function (data from *Arabidopsis thaliana*, as cited in the National Plant Genome Initiative 2014 report).

## Conclusions

SynFind fills the current gap of algorithm that performs syntenic gene queries and compiles matching set of genomic regions on-the-fly. SynFind identifies all syntenic regions to a given gene in a user-selected set of genomes, regardless of whether the gene is still present in that region. SynFind is powered by an algorithm that calculates synteny score between a pair of regions. Performance-wise, SynFind has higher sensitivity but lower purity compared with competing tools when validated against manually curated sets. Feature-wise, SynFind contains several key functions not typically found in existing systems (table 1). Integrated with the CoGe online platform and powered by the iPlant project, syntenic queries can now be performed in an interactive manner and retrieved for downstream analyses through SynFind in a scalable and reproducible manner. SynFind is an important tool for assessing genome dynamics including gene transpositions, impact of genome duplications, and correlation to functional changes across a set of related taxa of interest.

## Data Availability

SynFind is available for use through a web-based interface in CoGe. Data sets used in benchmarking SynFind with related tools are available on figshare with the following public DOI:

- Tang, Haibao (2015): SynFind supporting data: Benchmark on three curated syntenic gene sets. figshare. http://dx.doi.org/10.6084/m9.figshare.1589735 (last accessed November 30, 2015)
